# Survival prediction for patients with glioblastoma multiforme using a Cox proportional hazards denoising autoencoder network

**DOI:** 10.3389/fncom.2022.916511

**Published:** 2023-01-10

**Authors:** Ting Yan, Zhenpeng Yan, Lili Liu, Xiaoyu Zhang, Guohui Chen, Feng Xu, Ying Li, Lijuan Zhang, Meilan Peng, Lu Wang, Dandan Li, Dong Zhao

**Affiliations:** ^1^Key Laboratory of Cellular Physiology of the Ministry of Education, Department of Pathology, Shanxi Medical University, Taiyuan, Shanxi, China; ^2^College of Information and Computer, Taiyuan University of Technology, Taiyuan, China; ^3^Shanxi Provincial People's Hospital, Taiyuan, China; ^4^Department of Stomatology, Beijing Chaoyang Hospital, Capital Medical University, Beijing, China

**Keywords:** glioblastoma multiforme, deep learning, survival prediction, denoising autoencoder, time-dependent ROC curve, Cox proportional hazard regression

## Abstract

**Objectives:**

This study aimed to establish and validate a prognostic model based on magnetic resonance imaging and clinical features to predict the survival time of patients with glioblastoma multiforme (GBM).

**Methods:**

In this study, a convolutional denoising autoencoder (DAE) network combined with the loss function of the Cox proportional hazard regression model was used to extract features for survival prediction. In addition, the Kaplan–Meier curve, the Schoenfeld residual analysis, the time-dependent receiver operating characteristic curve, the nomogram, and the calibration curve were performed to assess the survival prediction ability.

**Results:**

The concordance index (C-index) of the survival prediction model, which combines the DAE and the Cox proportional hazard regression model, reached 0.78 in the training set, 0.75 in the validation set, and 0.74 in the test set. Patients were divided into high- and low-risk groups based on the median prognostic index (PI). Kaplan–Meier curve was used for survival analysis (*p* = < 2e-16 in the training set, *p* = 3e-04 in the validation set, and *p* = 0.007 in the test set), which showed that the survival probability of different groups was significantly different, and the PI of the network played an influential role in the prediction of survival probability. In the residual verification of the PI, the fitting curve of the scatter plot was roughly parallel to the *x*-axis, and the *p*-value of the test was 0.11, proving that the PI and survival time were independent of each other and the survival prediction ability of the PI was less affected than survival time. The areas under the curve of the training set were 0.843, 0.871, 0.903, and 0.941; those of the validation set were 0.687, 0.895, 1.000, and 0.967; and those of the test set were 0.757, 0.852, 0.683, and 0.898.

**Conclusion:**

The survival prediction model, which combines the DAE and the Cox proportional hazard regression model, can effectively predict the prognosis of patients with GBM.

## Introduction

Glioblastoma multiforme (GBM) is a tumor caused by the carcinogenesis of glial cells in the brain and spinal cord through the interaction of genetic and environmental factors (Weller et al., 2015; Xu et al., 2020). The potential risk factors include specific gene polymorphism, ionizing radiation, and virus infection. GBM may also cause increased intracranial pressure, edema, brain herniation, epilepsy, and mental symptoms as complications. Therefore, GBM is difficult to treat and frequently recurs, which results in high mortality (Poff et al., 2019; Peng et al., 2020). Due to individual differences and personalized therapy, the survival time of different patients with GBM shows high heterogeneity (Badve and Gökmen-Polar, 2015; Li et al., 2021). Studies have shown that the factors that affect the survival time of GBM include the morphology and degree of edema around the tumor, the degree of tumor necrosis, the overall morphology and size of the tumor, and whether the tumor has cystic changes.

Magnetic resonance imaging (MRI) is an important diagnostic tool for brain tumors (Takaya et al., 2021). It has been extensively applied in the diagnosis of brain tumors and some neurodegenerative diseases (such as Alzheimer's disease) (Wang et al., 2017; Yan et al., 2018) and has become a recognized imaging mode in the clinical treatment of GBM. Currently, conventional MRI assists in diagnosis, planning operative protocol, and monitoring disease progression and treatment response (Jenkinson et al., 2007). Radiomics is a technique that applies high-throughput computational approaches to extract quantitative features from images, such as MRI and PET. These features can be used to differentiate emerging cerebral lesions or predict the effect of different treatment options on patients with GBM. Radiomics also has the potential to non-invasively assess important prognostic and survival models (Lohmann et al., 2018; Conti et al., 2021; Schniering et al., 2022). By building the survival prediction model, clinicians can provide a reliable reference for each patient, formulate a therapeutic regimen, and evaluate the potency to alleviate patients' symptoms and improve the survival time.

Recently, deep learning-based models can learn more abstract features, reflecting more potential biological information. However, due to the complex patterns and sharpness, it is challenging to build a survival prediction model from medical images, especially using three-dimensional (3D) medical images. As the calculated amount arises, it becomes more challenging to construct an effective survival prediction model, while the advantages of deep learning are more prominent. Nie et al. (2019) used a multi-channel architecture of 3D convolutional neural networks (CNNs) for deep learning to extract high-level predictive features. Those deeply learned features and the limited demographic and tumor-related features are inputted into a support vector machine (SVM) to generate the final prediction result. The obtained multi-channel deep survival prediction framework can predict the survival time of patients with great accuracy. Lao et al. (2017) used the CNN_S model to extract deep features, then the least absolute shrinkage and selection operator (LASSO) Cox regression model was applied to construct a six-deep-feature signature. This study indicated the potential of deep imaging feature-based biomarkers in the preoperative care of patients with GBM. Zhu et al. (2016) used a deep CNN for survival analysis (DeepConvSurv) based on medical image data to prove that compared with the radiomics approach, this method has a significant performance improvement. Mobadersany et al. (2018) proposed the survival convolutional neural networks (SCNN) model, which performed deep learning to combine the functions of an adaptive machine-learning algorithm with a traditional survival model. This integrated model has high accuracy in predicting the survival rate of patients with GBM. Liu et al. (2019) proposed a 3D-deep CNN based on the attention mechanism to use multimodal MRI of GBM to predict survival. In this network, the attention module was incorporated into the deep-learning network to enhance the ability to express meaningful features while suppressing insignificant features. It involved a 3D volume of interest (VOI) from four modal MRIs as input, and the output was the risk value of each patient. The addition of the attention mechanism improved the predictive efficiency. Denoising autoencoder (DAE), an unsupervised deep-learning algorithm, is a random version of autoencoder formulation. It is designed to force the hidden layer of the autoencoder to capture more robust features. Wang et al. (2020) trained the autoencoder from a partially damaged (corrupted) input to rebuild a clean (repaired) input based on the basic principle. Thus, a good representation could be gained steadily from a damaged input, and the corresponding clean input could be restored. Tang et al. (2020) performed a multi-task CNN to select characteristics related to tumor genotype from preoperative multimodal MRI data to develop a tumor genotype and survival prediction model. However, whether the DAE methods can improve the extraction efficiency of survival features remains unknown.

In the present study, we built a convolutional DAE network with the loss function of the Cox proportional hazard regression model for the extraction of features for survival prediction. The DAE was applied to extract the features of the tumor area from the MRI of four modes and added into the risk prediction branch at the minimum feature matrix. The prognostic index (PI) was calculated from the risk scores of the Cox model and used for constructing a survival prediction model. This network can fully extract the features of the tumor region and build a more accurate prognostic model for patients with GBM.

## Materials and methods

### Data set and preprocessing

This article uses the MRI of 205 patients from the public dataset of the 2019 Multimodal Brain Tumor Segmentation (BraTS) Challenge containing four modalities. Clinical data for 100 of these patients were obtained from the Cancer Genome Atlas (TCGA) GBM project. All multimodal MRI scans are provided in NIfTI files (.nii.gz), and the four MRI modalities are T1, T2, T1ce, and Flair. According to the same annotation protocol, all imaging data sets were manually segmented by one to four evaluators according to the same annotation standards. Their annotations were approved by experienced neuroradiologists (Bakas et al., 2017a). The published dataset can be used freely on the premise of citing specific documents. All patients have survival time and survival status, and 100 of them have clinical data (age, sex, race, Karnofsky performance score (KPS), radiotherapy, chemotherapy).

**Figure 1:**
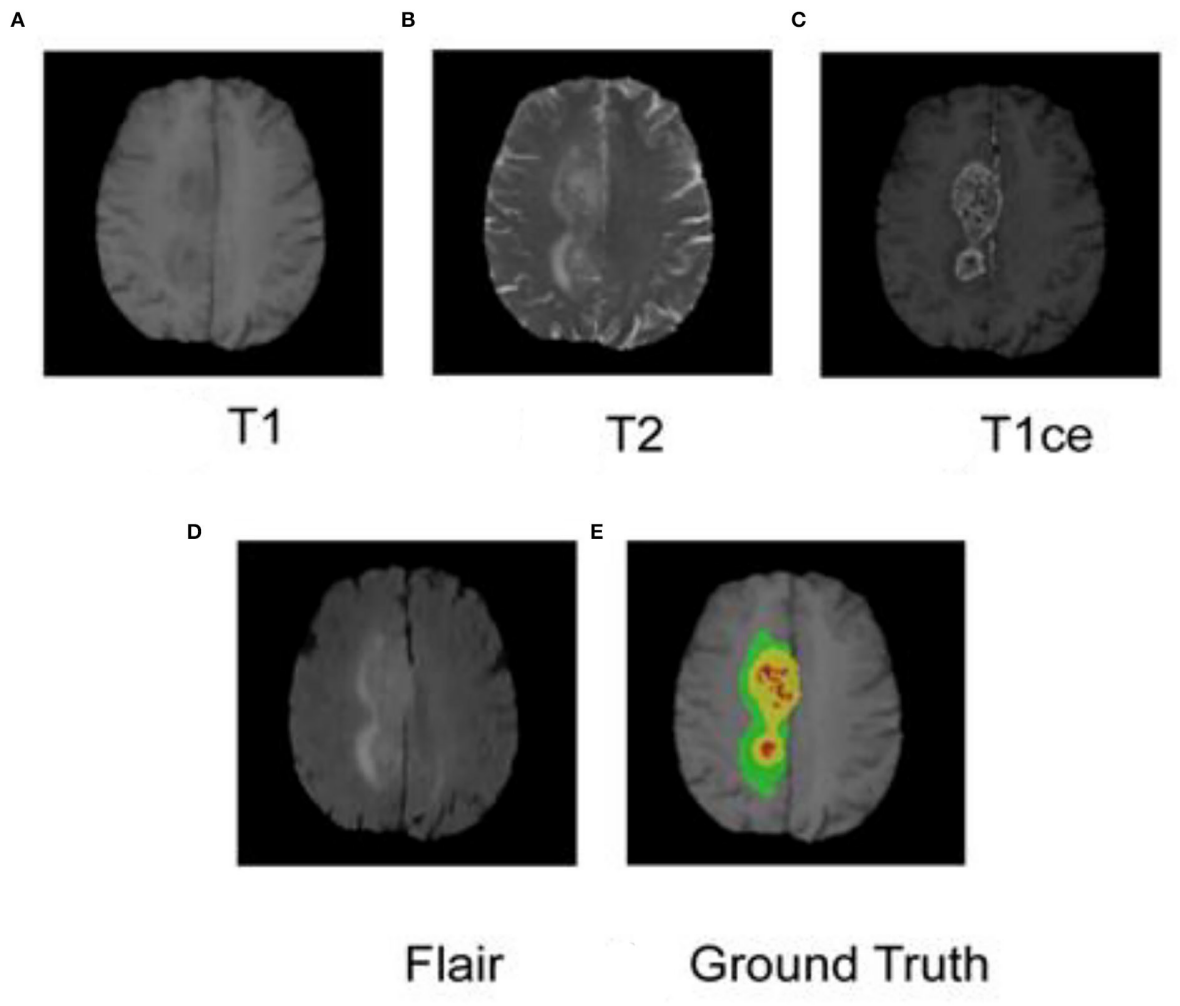
MRI and segmented labeling of the four modes of glioma. **(A)** T1, **(B)** T2, **(C)** T1ce, **(D)** Flair, and **(E)** Ground Truth. The labels in **(E)** are three nested regions, which are the whole tumor region (the green, yellow and red sets), the area of the tumor core (the yellow and red areas), and the Enhancing Tumor (ET) (the red area).

As shown in Figure 1, each case has four modes, namely T1, T2, T1ce, and Flair, and the specifications are 4 × 240 mm × 240 mm × 150 mm. At the same time, the data set also provides GBM labels manually segmented by multiple experts. The labels are three nested regions, which are the whole tumor region (the green, yellow, and red sets in Figure 1), the area of the tumor core (the yellow and red areas in Figure 1), and the enhancing tumor (ET) (the red area in Figure 1).

The public dataset provided by the competition has been partially preprocessed, including multimodal MRI data registered to the same spatial template, the image is resampled to 1 mm × 1 mm × 1 mm, and brain MRI also performed skull dissection. The dataset is provided by different scanning device configurations and institutions (Bakas et al., 2017b,c). Factors, such as the scanner itself and many unknown issues, can cause differences in brightness on MRI images, and the intensity value can vary within the same tissue, which is called a bias field. The bias field is a low-frequency smooth undesirable signal, which will cause unevenness in the MRI image. If the uncorrected bias field images are directly used for deep learning, it will affect the results of survival prediction. Therefore, before training the survival prediction model of GBM, it is necessary to perform offset field correction to minimize the influence of the offset field on survival prediction. This article uses ANTs N4BiasField Correction (Avants et al., 2009) to offset field correction. Then, the data of each mode is normalized separately. All the preprocessed data were compressed to H5 files. The training, validation, and test sets were randomly divided according to the index. At the same time, the data can be enhanced online (mirroring or rotating on different axes) during the input network.

### Network structure

**Figure 2:**
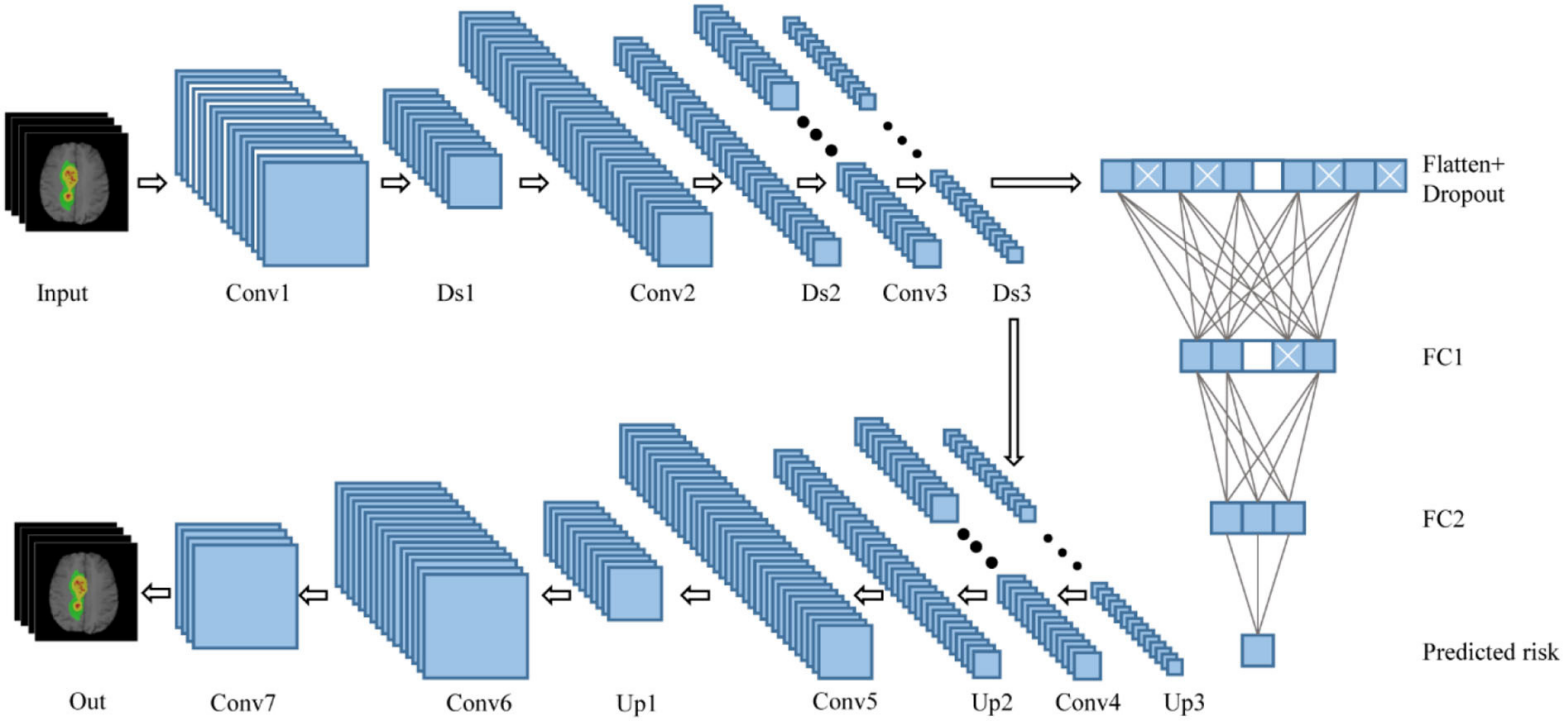
Network structure combining DAE and Cox. Conv, convolutional layer; Ds, downsampling layer; FC, fully connected layer; Up, upsampling layer.

In this study, the survival prediction model for GBM uses a convolutional DAE. Autoencoder (AE) is a deep neural network used for semi-supervised or unsupervised learning. The autoencoder can restore the output to the same as the input according to the deep features. After the autoencoder is trained, its output can replicate the input as much as possible. The network in this study is based on the DAE, a variant of the AE, which can realize the powerful functions of anti-noise and dimensionality reduction. The network structure of this article is shown in Figure 2. The self-encoder contains two parts: an encoder and a decoder. The encoder is expressed as φ*(x)*, and the input is the four modal MRI images of the GBM lesion area. After the input, the batch normalization layer and the drop out layer are added to randomly discard the image pixels to enhance the anti-noise and robustness of the network. The encoder includes three convolutions and downsampling to obtain the hidden characteristic matrix of the middle layer. Three convolutions generate 16, 32, and 64 feature maps, respectively, with a LeakyReLU activation behind each layer. The decoder is expressed as ψ*[*φ*(x)]*, which includes three convolutions and upsampling. Three convolutions generate 64, 32, and 16 feature maps, respectively, with a LeakyReLU activation behind each layer. The output layer of the decoder generates four feature maps through one convolution, followed by a sigmoid activation. Finally, it restores the features of the middle layer to the MRI images of the four modalities corresponding to the input. After the middle layer, the survival prediction branch was added, and the network was trained with the observed result data (survival/follow-up time). The feature matrix was flattened after the hidden layer feature matrix by Flatten. After flattening, there are a total of 65,536 features, using dense full connections to 1,024 features, randomly discarding (drop out) some neurons, and using dense full connections to 128 features, randomly discarding some neurons to prevent overfitting of the branching network. The feature corresponds to the 128-dimensional feature and the corresponding neural network weight. It also corresponds to the product of the independent variable and the partial regression coefficient in the Cox proportional hazard regression model.

### Loss function

A fusion of the reconstruction loss function and the Cox proportional hazard regression loss function is used in this network. In the reconstruction loss function of the convolution noise reduction autoencoder, the mean square error loss function is selected. It is defined as follows:


$$\begin{array}{l} L_{r}=\frac{1}{n}\overset{n}{\underset{i=1}{\sum }}||x_{i}-\psi \left(\phi \left(x_{i}\right)\right)|{|}^{2} \end{array}$$

where *n* represents the size of the sample. Minimizing the reconstruction loss function ensures that the hidden layer in the DAE can effectively learn the potentially valuable features in GBM MRI.

Cox proportional hazards regression model with survival outcome and survival time as dependent variables can simultaneously analyze the impact of multiple factors on survival time. To ensure that the hidden layer can effectively process censored data and extract features that are incredibly relevant and robust to survival, the Cox proportional hazard regression model is applied to create the loss function after densely connected in the middle layer. At the same time, the Cox proportional hazard regression model can better correct the influence of multiple confounding factors on the results. The Cox proportional hazard regression model is defined as follows:


$$\begin{array}{l} \log\frac{h_{i}\left(t\right)}{h_{0}\left(t\right)}=\beta_{1}z_{i1}+\beta_{2}z_{i2}+\ldots \beta_{P}z_{iP}, \end{array}$$

where *h*_*i*_(*t*) represents the risk function of patient *i* and is the probability of the subject's death at time *t*. *h*_0_(*t*) is a baseline risk level, and the *h*_*i*_(*t*) (i = 1,..., n) risk functions of all patients at different times are compared with it. The critical assumption of the Cox prognostic model is that the hazard ratio *h*_*i*_(*t*)/*h*_0_(*t*) is constant for time. The natural logarithm of the ratio is the weighted sum of multiple predictors (here represented by *z*_*i*1_,..., *z*_*ip*_), and the weight coefficients are represented by β_1_,..., β_*p*_. These coefficients are estimated by maximizing the partial likelihood function of Cox's proportional hazards:


$$\begin{array}{l} \log\zeta \left(\beta \right)=\overset{n}{\underset{i=1}{\sum }}\delta_{i}\left\{\beta^{\prime }z_{i}-\log\underset{j\in R\left(t_{i}\right)}{\sum }e^{\beta^{\prime }z_{j}}\right\} \end{array}$$

Among them, *z*_*i*_ is the vector used to predict patient *i*, δ_*i*_ is an indicator of patient i's survival status (0 = survival or censorship, 1 = death), and *R*(*t*_*i*_) represents the risk vector set of patient *i*. This study applies the loss function to the survival prediction model, which is defined as follows:


$$\begin{array}{l} L_{s}=-\overset{n}{\underset{i=1}{\sum }}\delta_{i}\left\{W^{\prime }\phi \left(x_{i}\right)-\log\underset{j\in R\left(t_{i}\right)}{\sum }e^{W^{\prime }\phi \left(x_{j}\right)}\right\} \end{array}$$

Among them, *W*, represents the weight vector of the final output of the survival prediction branch and ϕ(*x*_*i*_) represents the feature vector of the prediction branch. The multiplication of the two is the patient's risk prediction, which is the natural logarithm of the hazard ratio.

In this study, the loss function of the prognostic model is composed of the reconstruction loss function and the loss function of the Cox proportional hazard regression model, which is defined as follows:


$$\begin{array}{l} L_{hybrid}=\alpha L_{r}+\beta L_{s}=\alpha \left[\frac{1}{n}\overset{n}{\underset{i=1}{\sum }}||x_{j}-\psi \left(\phi \left(x_{i}\right)\right)|{|}^{2}\right]\\ +\beta \left[-\overset{n}{\underset{i=1}{\sum }}\delta_{i}\left\{W^{\prime }\phi \left(x_{i}\right)-\log\underset{j\in R\left(t_{i}\right)}{\sum }e^{W^{\prime }\phi \left(x_{j}\right)}\right\}\right] \end{array}$$

Among them, α and β are the reconstruction loss functions, and the loss function of the Cox proportional hazard regression model is the weight coefficient. For convenience, the sum of α and β is set to 1.

### Survival prediction model training

A total of 205 cases of MRI and survival data from BraTS2019 containing four modalities are used in this study. These cases are divided into 143 cases of the training set, 31 cases of the validation set, and 31 cases of the test set. The result of the segmentation of the lesion area is used as the input to the model. The DAE and the Cox proportional hazard regression loss functions are used to extract the multimodal image data of GBM. Finally, the PI of each individual is not affected by time changes. The data are randomly scrambled before entering the network. The batch size in the input network is 26, and each batch is sorted from short to long in the order of survival time. The ratio of the reconstructed loss function and the loss function of the Cox proportional hazard model was determined to be 7:3 after several experiments. The model has been optimized by Adam. The initial learning rate is 5e-4, and the epoch is 200. The learning rate will decay by 0.5 after 10 epochs if the validation loss is not improving. The maximum number of iterations of training is set to 300.

### Survival prediction model evaluation

In this study, we used the concordance index (C-index) and the accuracy to evaluate the model's performance. The C-index was obtained by combining the PI with the individual's survival time and status and calculated by the R “Hmisc” package. The accuracy is calculated based on a three-category classification. We define the survival days < 300 as a high-risk group, from 300 to 450 as a mid-risk group, and more than 450 as a low-risk group (Bakas et al., 2018), and divided the PI evenly into three parts in the training set, which was defined as low-, medium-, and high-risk groups. To assess the performance of the survival prediction model proposed in this study, its accuracy is compared with three methods: *post hoc* (Hermoza et al., 2021), random forest regressor (RFR) (Agravat and Raval, 2019), and a survival prediction model using neural networks (Wang et al., 2019).

The survival prediction model was constructed by combining the PI (produced by the network), the survival time, and the survival status of each individual. All patients were divided into high- and low-risk groups according to the median of the PI. Then, we applied a Kaplan–Meier survival analysis and the log-rank test to evaluate the model's stratification ability. This study used Schoenfeld residual method to verify whether the PI predicted by the network is time-dependent (Zhang et al., 2018). A Lowess (locally weighted scatterplot smoothing) smoothing function was used for fitting to obtain the smooth curve of Schoenfeld residuals and time. The correlation between Schoenfeld residuals and time rank was tested to investigate the independence between residuals and time. If the *p*-value of the test is more than 0.05, it proves that the linear relationship between residual and time is not significant, which further shows that the PI does not depend on time changes. To evaluate the predictive ability of the PI predicted by the network and whether the predictive power of the PI decreases over time, we constructed the time-dependent receiver operating characteristic (ROC) curve for evaluation. There were stages every 200 days, and fewer patients survived 800 days. The four stages were evaluated in this study to reduce the impact of the small patient sample number in the later stage.

### Nomogram construction and evaluation

In addition, combined with clinical risk factors such as age, gender, race, KPS, radiotherapy, and chemotherapy, a nomogram was built on the predictive model for the training set to predict overall survival (OS). The calibration curve was constructed to analyze the diagnostic performance of the nomogram in the training set, validation set, and test set.

## Results

### Performance of survival prediction models

In this study, the consistency index of the survival prediction model that combines the DAE and the Cox model reached 0.78 in the training set, 0.75 in the validation set, and 0.74 in the test set. As shown in Table 1, the accuracy was used to evaluate the performance of different prognostic models. In the test set, the accuracy of our proposed model reaches 0.548, which is 0.3% lower than the model proposed by Wang et al. (2019). However, in the training set and validation set, our proposed method achieves a higher accuracy value than the other three survival prediction models in Table 1. Therefore, our proposed method has a slight advantage. The model proposed in this study could extract the robust features related to survival prediction from the multimodal MRI of GBM lesions and could process censored data.

**Table 1:** Comparison of the results of different prognostic models.

**Model**	**Training set**	**Verification set**	**Test set**
Hermoza et al. (2021)	0.548	0.517	-
Agravat and Raval (2019)	0.554	0.517	-
Wang et al. (2019)	0.515	0.448	0.551
DAE + Cox	0.664	0.613	0.548

### Confirm the validity of the PI

**Figure 3:**
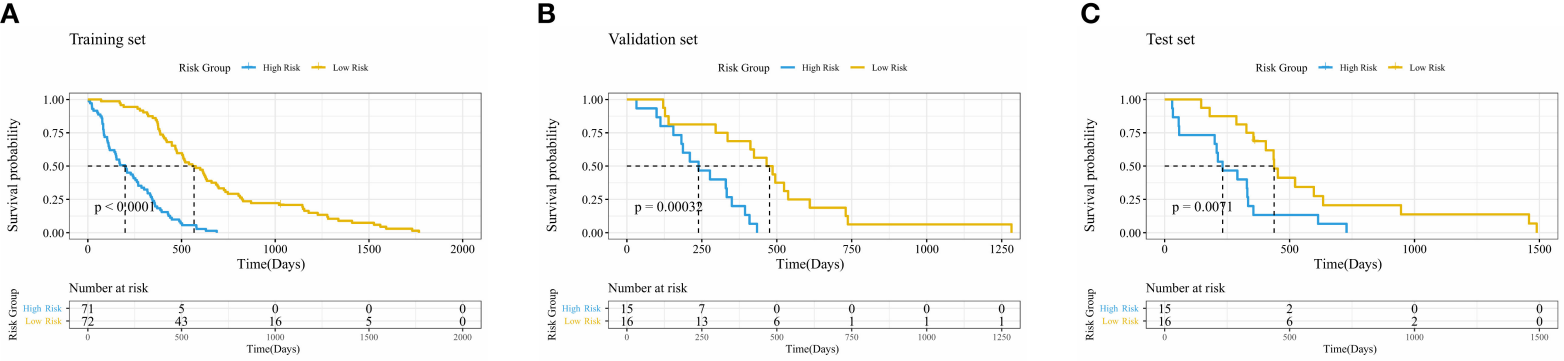
Kaplan–Meier curve of risk grouping of the training set **(A)**, validation set **(B)**, and test set **(C)**.

The Kaplan–Meier survival analysis with log-rank test results is shown in Figure 3, where *p* = < 2e-16 in the training set, *p* = 3e-04 in the validation set, and *p* = 0.007 in the test set. The *p*-value of log-rank test results was < 0.05 in all three sets, which proved that the survival probability of different groups divided by PI was significantly different. It also suggested that the PI predicted by the network had an influential role in predicting survival probability.

### PI independence with time

**Figure 4:**
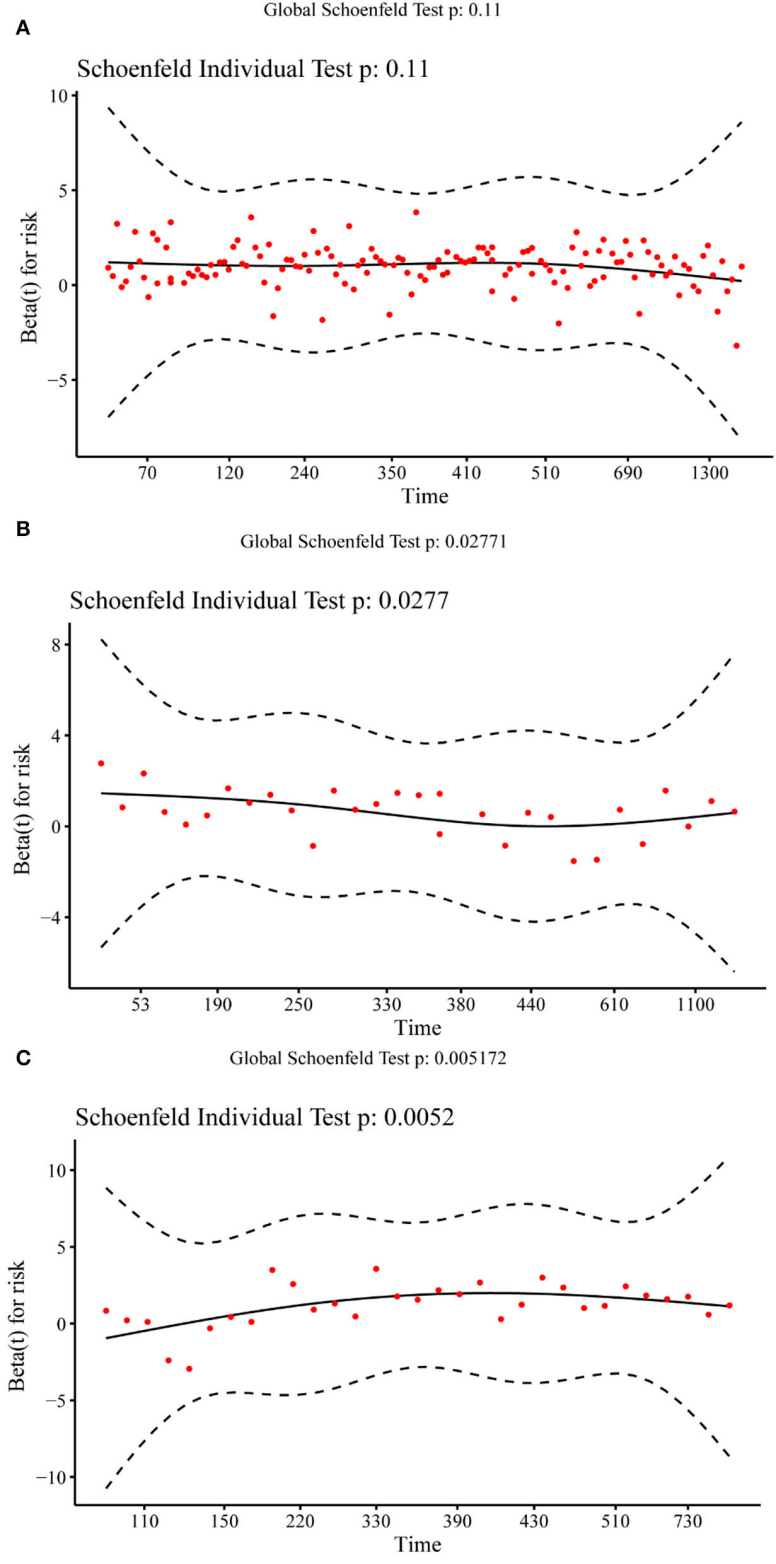
Schoenfeld residual plot of the prognostic index in the training set **(A)**, validation set **(B)**, and test set **(C)**.

Figure 4 shows the Schoenfeld residual plot of the PI in the training set, validation set, and test set. In the residual check of the PI, the fitting curve of the scatter plot was roughly parallel to the *x*-axis. In addition, the *p*-value of the test was 0.11, more than 0.05, proving that the PI and survival time were independent of each other and indicating that the survival prediction ability of the PI was less affected by time.

### Time-dependent accuracy analysis of the model

**Figure 5:**
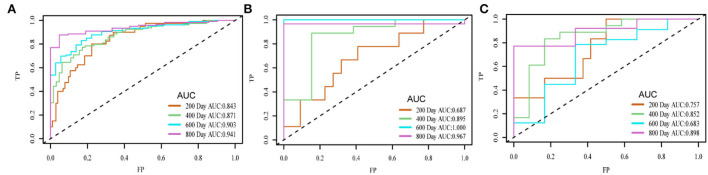
Time-dependent ROC of the training set **(A)**, validation set **(B)**, and test set **(C)**.

Figure 5 shows the time-dependent ROC of the training set, validation set, and test set. The area under the curve (AUC) value of each period of the three sets was relatively high. The AUC in the last stage was relatively high because almost all high-risk patients had died. On the three sets, the attenuation of AUC was minimal, which proved that the predictive ability of the PI attenuates to a small extent with time.

### Development and validation of the nomogram

**Figure 6:**
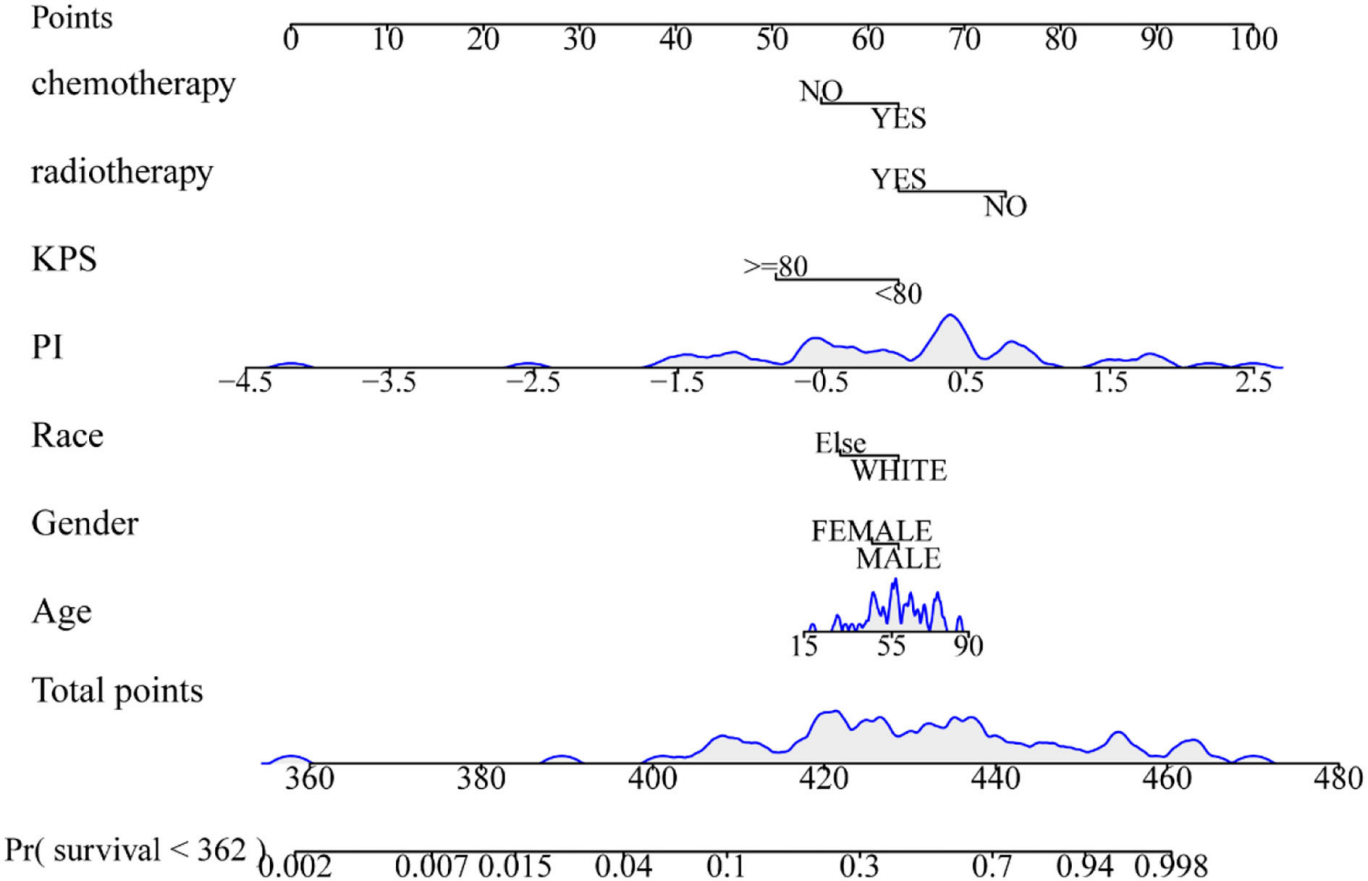
Radiomics nomogram for overall survival of patients with GBM. The shaded part indicates the distribution status and probability density of the patients.

**Figure 7:**
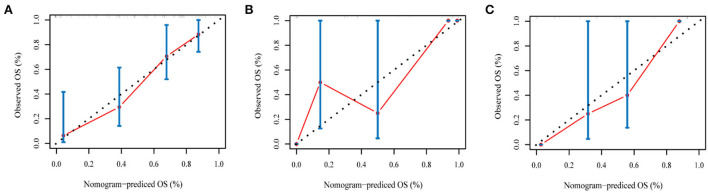
The calibration curves of the radiomics nomogram in the training set **(A)**, validation set **(B)**, and test set **(C)** of the overall survival of patients with GBM. The calibration curves depict the calibration of the nomogram in terms of the agreement between the predicted risk and the observed survival.

The radiomics nomogram incorporating the PI and seven clinical factors was constructed based on the multivariate Cox regression (Figure 6). The figure represents the survival prediction model for patients with GBM. The consistency index of the nomogram model reached 0.79 in the training set, 0.74 in the validation set, and 0.75 in the test set. Calibration curves (Figure 7) showed that the predicted OS of the nomogram was closely aligned with the observed OS in the training set, validation set, and test set.

## Discussion

The structure of brain GBM is complex, and tumor tissue mainly includes edema, necrosis, and tumor core. In addition, the traditional extraction of imaging features is limited, and many deeper features of brain GBM cannot be effectively extracted. Autoencoder is an unsupervised learning technology that applies neural networks for representational learning to overcome the heterogeneity of individual tumors and contribute to the noise reduction of images. These are the advantages of traditional CNN networks.

Prognostic models are designed to assess the impact of specific prognostic factors on events of interest over time and predict the risk of future possibilities for new patients. The cure rates of patients with GBM are inferior, and the survival rates are often worse or even almost impossible to cure. Accurate prediction of survival probability is essential for the treatment of patients. Therefore, it is urgent to develop a prognostic model to assess prognostic variables (Cheng et al., 2013; Royston and Altman, 2013; Yeh et al., 2016). The most popular prognostic model is the Cox proportional risk regression model proposed by Cox (1972), a semi-parametric regression model. Cox regression provides the direction of DAE learning and can capture more features related to survival. It has unique advantages in constructing a survival prediction model, which can analyze the impact of multiple factors on survival and process deleted data. It can obtain the risk level, which is different from the traditional loss function used for survival prediction. However, it is assumed that the linear logarithmic risk function is too simple for clinical survival data. As a result, many researchers have proposed non-linear risk models to fit survival data as much as possible, such as Cox regression based on neural networks (Cox, 1972) and multi-task survival analysis learning (Li et al., 2016).

However, it is challenging to build a prediction model with all the features, and it is prone to overfitting, which would lead to inaccurate prediction results. Hence, it is necessary to screen out the crucial features and eliminate the insignificant ones, which led to the later development of Lasso-Cox (Zhang and Lu, 2007), Ridge-Cox (Vinzamuri and Reddy, 2013), and EN-Cox (Simon et al., 2011) models. In this study, we proposed a hypothesis of a survival prediction model combining the DAE and Cox proportional hazards regression model. The empirical results show that the model can better predict the survival of patients with GBM. Through this model, we could obtain not only the compelling image features but also the features that can represent survival.

To evaluate the predictive power of the PI derived from network prediction, we applied time-dependent ROC curve analysis, which was constructed at different survival time points. The result showed that the prediction ability of the PI was practical with the increase in time. On the training set, validation set, and test set, the attenuation of AUC is minimal, which proves that the predictive ability of the PI attenuates to a small extent with time. In the Kaplan–Meier survival and log-rank test, the *p*-value was < 0.05 in the training set, validation set, and test set, proving that higher and lower PI values were significantly different. It also confirmed that the PI of the network played an influential role in predicting survival probability (Cui et al., 2020). In the residual verification of the PI, the fitting curve of the scatter plot was roughly parallel to the x-axis. The *p*-value of the test was 0.11, more than 0.05, proving that the PI and survival time were independent of each other and indicating that the survival prediction ability of the PI was less affected by time (Kwon et al., 2015). Previous studies (Sun et al., 2015; Chen et al., 2021) have confirmed that clinical factors, such as age, gender, and KPS, are important variables related to the prognosis of GBM. We constructed a nomogram based on the PI and clinical factors. The calibration plots for the probabilities of OS showed good agreement between the predicted OS by nomogram and the actual OS of patients with GBM. The results suggested the accuracy of the nomogram and further indicated that the nomogram can accurately predict the prognosis of patients with GBM (Liu et al., 2020). Compared with PI, the predictive performance of the nomogram is improved.

Despite the promising results, this study still has several limitations. This study is retrospective, and only the Cancer Imaging Archive (TCIA) database was used. In the future, a multicenter study is needed to richly assess the generalization ability of the survival prediction model (Lao et al., 2017). In addition, the survival prediction model also can be added with TNM stage classification tasks that might contribute to survival prediction in the future and make better adjustments to the consistency index of survival prediction.

This study proposes a GBM survival prediction model based on DAE and Cox proportional hazard regression loss function. The survival prediction branch is added, and the Cox proportional hazard regression model is used as the auxiliary loss function based on DAE. The results indicate that the model can predict the prognosis of patients with GBM well.

## Data availability statement

The original contributions presented in the study are included in the article/supplementary material, further inquiries can be directed to the corresponding author/s.

## Ethics statement

Ethical review and approval was not required for the study on human participants in accordance with the local legislation and institutional requirements. Written informed consent from the patients/participants or patients/participants' legal guardian/next of kin was not required to participate in this study in accordance with the national legislation and the institutional requirements.

## Author contributions

TY conceived the study, designed the experiments, analyzed the data, and wrote the manuscript. DL and DZ edited the manuscript. ZY, LL, XZ, GC, FX, YL, and LZ supervised data analysis. MP and LW performed the statistical analyses. All authors accessed the study data and reviewed and approved the final manuscript version.
